# Comprehensive Analysis of the Molecular Characteristics and Prognosis value of AT II-associated Genes in Non-small Cell Lung Cancer

**DOI:** 10.1155/2022/3106688

**Published:** 2022-09-26

**Authors:** Liping Ren, Xiaoxia Wen, Mujiexin Liu, Yao Xiao, Ping Leng, Huaichao Luo, Pei Tao, Lei Xie

**Affiliations:** ^1^School of Healthcare Technology, Chengdu Neusoft University, Chengdu, China; ^2^Chongqing Key Laboratory of Sichuan-Chongqing Co-construction for Diagnosis and Treatment of Infectious Diseases Integrated Traditional Chinese and Western Medicine, College of Medical Technology, Chengdu University of Traditional Chinese Medicine, Chengdu, China; ^3^Ineye hospital of Chengdu University of Traditional Chinese Medicine, Chengdu, China; ^4^Department of clinical laboratory, Sichuan Cancer Hospital & Institute, Sichuan Cancer Center, School of Medicine, University of Electronic Science and Technology of China, Chengdu, Sichuan, China; ^5^Chengdu Women's and Children's Central Hospital, School of Medicine, University of Electronic Science and Technology of China, Sichuan 611731, China; ^6^The Sichuan Provincial Key Laboratory for Human Disease Gene Study and Department of Laboratory Medicine, Sichuan Provincial People's Hospital, School of Medicine, University of Electronic Science and Technology of China, Chengdu, China

## Abstract

Alveolar type II (AT II) is a key structure of the distal lung epithelium and essential to maintain normal lung homeostasis. Dedifferentiation of AT II cells is significantly correlated with lung tumor progression. However, the potential molecular mechanism and clinical significance of AT II-associated genes for lung cancer has not yet been fully elucidated. In this study, we comprehensively analyzed the gene expression, prognosis value, genetic alteration, and immune cell infiltration of eight AT II-associated genes (AQP4, SFTPB, SFTPC, SFTPD, CLDN18, FOXA2, NKX2-1, and PGC) in Nonsmall Cell Lung Cancer (NSCLC). The results have shown that the expression of eight genes were remarkably reduced in cancer tissues and observably relating to clinical cancer stages. Survival analysis of the eight genes revealed that low-expression of CLDN18, FOXA2, NKX2-1, PGC, SFTPB, SFTPC, and SFTPD were significantly related to a reduced progression-free survival (FP), and low CLDN18, FOXA2, and SFTPD mRNA expression led to a short postprogression survival (PPS). Meanwhile, the alteration of 8 AT II-associated genes covered 273 out of 1053 NSCLC samples (26%). Additionally, the expression level of eight genes were significantly correlated with the infiltration of diverse immune cells, including six types of CD4+T cells, macrophages, neutrophils, B cells, CD8+ T cells, and dendritic cells. In summary, this study provided clues of the values of eight AT II-associated genes as clinical biomarkers and therapeutic targets in NSCLC and might provide some new inspirations to assist the design of new immunotherapies.

## 1. Introduction

Lung cancer is one of the most commonly diagnosed cancers and the leading cause of cancer-related death in the world [1–3]. Nonsmall cell lung cancer (NSCLC) is one of the most majorly types of lung cancer (approximately 85%), mainly including lung adenocarcinoma (LUAD) and lung squamous cell carcinoma (LUSC) [2, 4–7]. Studies have demonstrated that the average five-year survival rate of NSCLC patients is 15% [1, 8]. This poor survival rate is attributable to many factors, such as delays in the diagnosis of lung cancer and limited therapies currently available [9, 10]. Over the past decade, with the improvement of treatment technologies and the emergence of the era of precision radiotherapy, the diagnosis, and treatment of lung cancer have been improved to a certain extent [9, 11–16]. Despite advances in treatment, the overall prognosis for NSCLC has not yet improved significantly.

The alveolar cells are mainly composed of alveolar type I (AT I) cells and alveolar type II (AT II) cells [17, 18]. There into, AT II is a key structure of the distal lung epithelium and has a secretory function that is essential to maintaining normal lung homeostasis [19]. In recent years, there is currently substantial evidence showing that AT II and AT II-associated genes are significantly related to the occurrence and development of multiple diseases [20]. One of the pathological features of the idiopathic pulmonary fibrosis (IPF) lung is the senescence of AT II [21, 22]. AT II is also involved in the occurrence and development of Chronic obstructive pulmonary disease (COPD) through the upregulated expression of many anti- or proinflammatory genes, including genes encoding oxygenase 2 (HO-2) and inducible nitric oxidase (iNOS) [20]. Importantly, several studies have also shown that AT II plays a crucial role in the oncogenesis of lung cancer [8, 23]. Single-cell RNA sequencing of lung cancer tissues revealed that some AT II-associated genes expressed differently in the lung cancer cells, including aquaporin 4 (AQP4), surfactant pulmonary associated protein B (SFTPB), surfactant pulmonary associated protein C (SFTPC), surfactant pulmonary associated protein D (SFTPD), claudin 18 (CLDN18), forkhead box A2 (FOXA2), NKX homeobox-1 gene (NKX2-1), and pepsinogen C (PGC) [24]. However, the potential values of these AT II cell-related genes in NSCLC have not been fully clarified.

Therefore, in this study, we performed a comprehensive analysis, including analysis of gene expression, prognosis value, genetic alteration, and immune cell infiltration of these eight AT II-associated genes in two subtypes of NSCLC (LUAD and LUSC). It aims to provide clinicians with additional information to assess and adjust the diagnostic methods and treatment options of NSCLC patients.

## 2. Materials and Methods

### 2.1. Oncomine

Oncomine database is a publicly accessible online cancer microarray database. (http://www.oncomine.org/), which provides a genome-wide expression analysis for a wide variety of tumor types [25]. In this study, it was utilized to analyze the transcription levels of AT II-associated genes in NSCLC tissues and their corresponding adjacent normal control samples. The *p*value < 0.05 (Student's *t*-test), fold change of 2, and gene rank in the top 10% were set as the significance.

### 2.2. Gene Expression Profiling Interactive Analysis (GEPIA)

GEPIA (http://gepia.cancer-pku.cn/index.html) is a newly developed interactive web server for analyzing the RNA sequencing expression data of 9736 tumors and 8587 normal samples from the TCGA and Genotype-Tissue Expression dataset [26]. GEPIA offers customizable functions such as tumor/normal differential expression analysis, patient survival analysis, similar gene detection, correlation analysis, and dimensionality reduction analysis. The Student's *t*-test was used to generate a *p* value (*p* value < 0.01). In this study, we performed the pathological type and stage analysis of eight AT II-associated genes using the “LUAD” and “LUSC” datasets (one-way ANOVA).

### 2.3. Kaplan-Meier Plotter

Kaplan-Meier Plotter (https://kmplot.com/analysis/) is a useful prognostic biomarker assessment tool that can assess the effect of 54 k genes on survival in 21 cancer types [27]. In this study, LUAD and LUSC patients were split into high and low-expression groups based on median values of AT II-associated genes expression and analyze their prognostic value in LUAD and LUSC regarding OS (overall survival), FP (first progression), and PPS (postprogression survival). The hazard ratio with 95% confidence intervals and log rank *p* value was calculated (*p* value < 0.05).

### 2.4. cBioPortal

cBioPortal (http://www.cbioportal.org/) is a comprehensive web resource that could visualize and analyze multidimensional cancer genomics data [28, 29]. In this study, we analyze the AT II-associated genes' multiple alterations for LUAD (TCGA, Pan-Cancer Atlas) and LUSC (TCGA, Pan-Cancer Atlas), which contained mutations, structural variants, and copy-number alterations.

### 2.5. STRING

STRING (https://string-db.org/) is a database of known and predicted protein–protein interactions (PPI) [30]. In this study, we conducted associations among the PPI network of AT II-associated genes to explore the role of AT II-related genes' coexpressed genes with STRING.

### 2.6. GeneMANIA

GeneMANIA (http://www.genemania.org) is a useful website that can find information on protein–protein, protein–DNA, and genetic interactions, pathways, reactions, gene and protein expression data, protein domains, and phenotypic screening profiles [31]. In this study, we used it to measure the attribute above for AT II-associated genes.

### 2.7. Timer

Timer web server (https://cistrome.shinyapps.io/timer/) is a comprehensive resource for systematic analysis of the infiltration of different immune cells and their clinical impact across diverse cancer types [32]. In this study, we use the “Gene module” and “Survival module” to explore the correlation of eight AT II-associated gene levels and the immune cell infiltration.

## 3. Results

### 3.1. Differential Expression of AT II-associated genes in Patients With NSCLC

**Figure 1:**
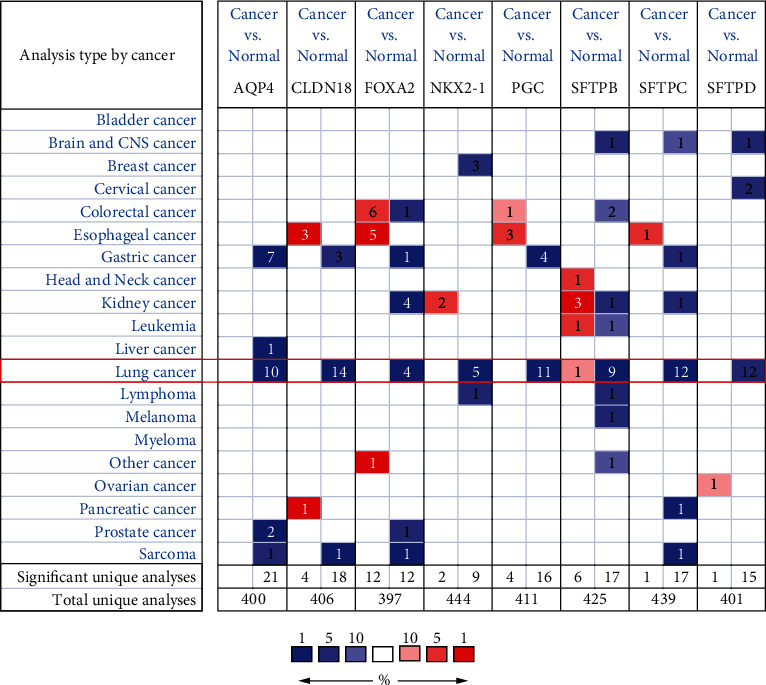
Expression of AT II-associated genes in different cancer types (Oncomine). The graphic demonstrates the numbers of datasets with statistically significant alterations in the mRNA expression of the target gene: upregulated (red) and downregulated (blue). The following criteria were used: *p* value of 0.05, fold change of 2, and gene rank of 10%. As shown in the green frame, expression levels of AQP4, SFTPB, SFTPC, SFTPD, CLDN18, FOXA2, NKX2-1, and PGC were significantly reduced in lung cancer.

**Figure 2:**
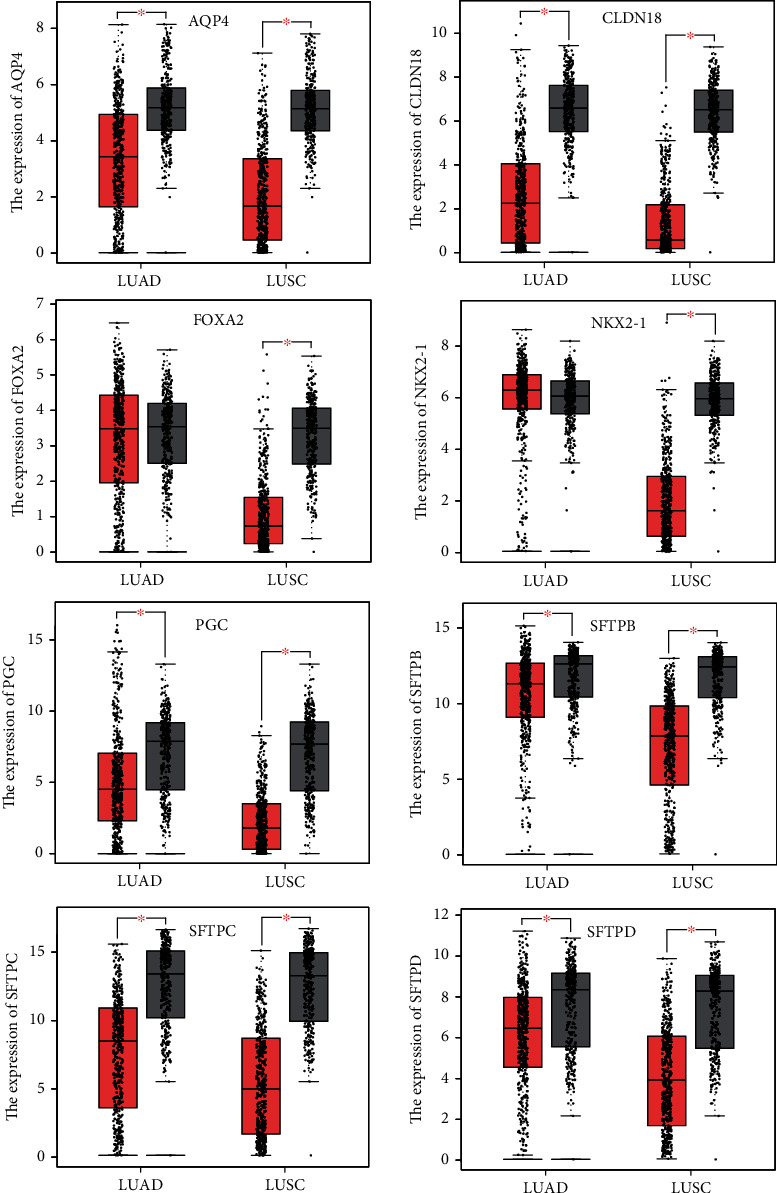
The expressions of the AT II-associated genes in LUAD and normal tissues and LUSC and normal tissues (GEPIA). The results indicated that AQP4, CLDN18, PGC, SFTPB, SFTPC, and SFTPD were lower in LUAD tissues than in normal tissue, and AQP4, CLDN18, FOXA2, NKX2-1, PGC, SFTPB, SFTPC, and SFTPD were lower in the LUSC tissues than the normal tissues. ^∗^*p* < 0.01.

Firstly, we explored the expression levels of 8 AT II-associated genes in lung cancer and normal lung tissues using the ONCOMINE database. As the results shown in Figure 1, the expression levels of AQP4, CLDN18, FOXA2, NKX2-1, PGC, SFTPB, SFTPC, and SFTPD were all remarkably reduced in lung cancer vs. normal tissues in multiple datasets. Furthermore, we compared the expressions of the eight AT II-associated genes in LUAD (483 LUAD and 347 normal tissues) and LUSC (486 LUSC and 338 normal tissues) by GEPIA. The results in Figure 2 indicated that the expression of AQP4, CLDN18, PGC, SFTPB, SFTPC, and SFTPD decreased in LUAD tissues and AQP4, CLDN18, FOXA2, NKX2-1, PGC, SFTPB, SFTPC, and SFTPD decreased in in the LUSC tissues. We also contrasted the relative expression levels of eight AT II-related genes in LUAD and LUSC tissues and determined that among all the factors we evaluated, SFTPB was the highest expression in both LUAD and LUSC (Figure S1). Taken together, our results showed that the expressions of AQP4, CLDN18, FOXA2, NKX2-1, PGC, SFTPB, SFTPC, and SFTPD were significant decrease in in NSCLC.

### 3.2. Correlation Between AT II-associated genes and Tumor Stages of NSCLC Patients

**Figure 3:**
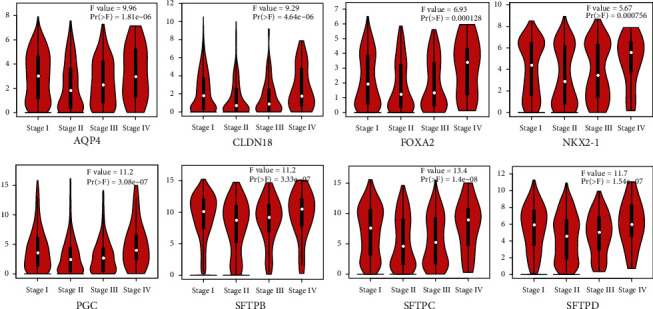
Correlation between expression of AT II-associated genes and tumor stage in NSCLC (GEPIA). The expressions of AQP4, CLDN18, FOXA2, NKX2-1, PGC, SFTPB, SFTPC, and SFTPD were distinctly related to patients' individual cancer stages (one-way ANOVA).

Lung cancer is divided into four stages according to the disease progression. As the condition develops, the patient's physiology and physical condition will also constantly change. Therefore, we assessed the correlation between the expression of AT II-associated genes and the patients' pathological cancer stages of LUAD and LUSC patients by using GEPIA. We found that the expression of all eight AT II-associated genes are significant correlated with pathological stage of NSCLC (Figure 3). AQP4 (*p* = 1.81e − 06), CLDN18 (*p* = 4.64e − 06), FOXA2 (*p* = 1.28e − 04), NKX2-1(*p* = 7.56e − 04), PGC (*p* = 3.08e − 07),SFTPB (*p* = 3.33e − 07), SFTPC (*p* = 1.4e − 08), and SFTPD (*p* = 1.54e − 07) show that the AT II-associated genes were inclined to high expression in NSCLC patients with advanced cancer stages (Figure 3). These data suggested that the 8 AT II-associated might play a significant role in the tumorigenesis and progression of NSCLC.

### 3.3. Prognostic Features of AT II-associated genes in Patients with Lung Cancer

**Figure 4:**
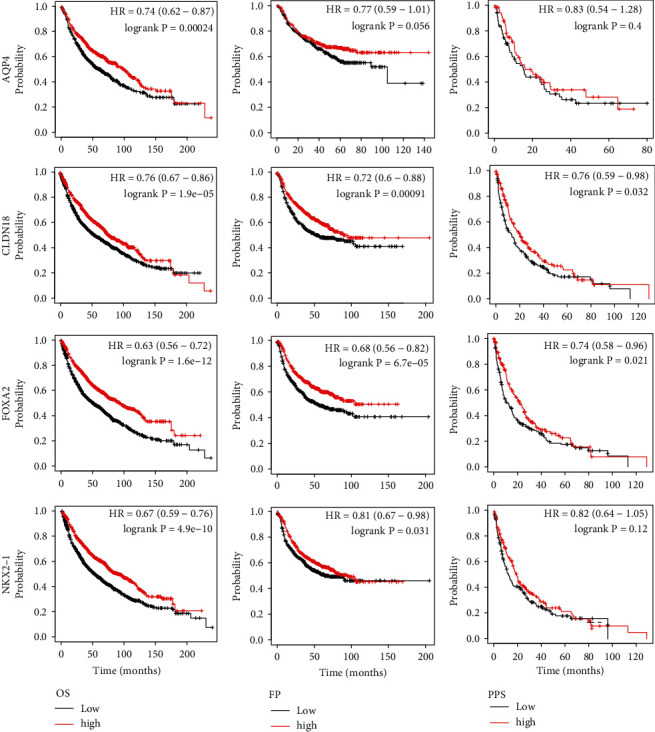
Prognostic value of AT II-associated genes (AQP4, CLDN18, FOXA2, and NKX2-1) in LUAD and LUSC (Kaplan-Meier plotter).

To analyze the prognostic values of AT II-associated genes in NSCLC patients, we assessed the correlation between these genes and overall survival (OS), progression-free survival (FP), and postprogression survival (PPS) using Kaplan-Meier plotter (Table 1). The results shown in Figure 4 and Figure S2 are the low-expression of genes including AQP4 (HR = 0.74, *p* = 2.40*e* − 04), CLDN18 (HR = 0.76, *p* = 1.9*e* − 05), FOXA2 (HR = 0.63, *p* = 1.6*e* − 12), NKX2-1 (HR = 0.67, *p* = 4.9*e* − 10), PGC (HR = 0.69, *p* = 1*e* − 08), SFTPB (HR = 0.67, *p* = 6.3*e* − 10), SFTPC (HR = 0.81, *p* = 1.40*e* − 03), and SFTPD (HR = 0.66, *p* = 1.6*e* − 10) were significantly associated with low OS. And the low-expression of CLDN18 (HR = 0.72, *p* = 9.10*e* − 04), FOXA2 (HR = 0.68, *p* = 6.7*e* − 05), NKX2-1 (HR = 0.81, *p* = 3.10*e* − 02), PGC (HR = 0.7, *p* = 2.40*e* − 04), SFTPB (HR = 0.82, *p* = 4.80*e* − 02), SFTPC (HR = 0.82, *p* = 4.00*e* − 02), and SFTPD (HR = 0.68, *p* = 6.2*e* − 05) were significantly related to a reduced FP. Low-expression of CLDN18 (HR = 0.98, *p* = 3.20*e* − 02), FOXA2 (HR = 0.74, *p* = 2.10*e* − 02), and SFTPD (HR = 0.96, *p* = 2.10*e* − 02) apparently led to a short PPS. Moreover, no significant difference was found between the AT II-associated genes and disease-free survival **(**DFS) in NSCLC patients (Table 1).

### 3.4. Genetic Alteration and PPI Analyses of AT II-associated genes

**Figure 5:**
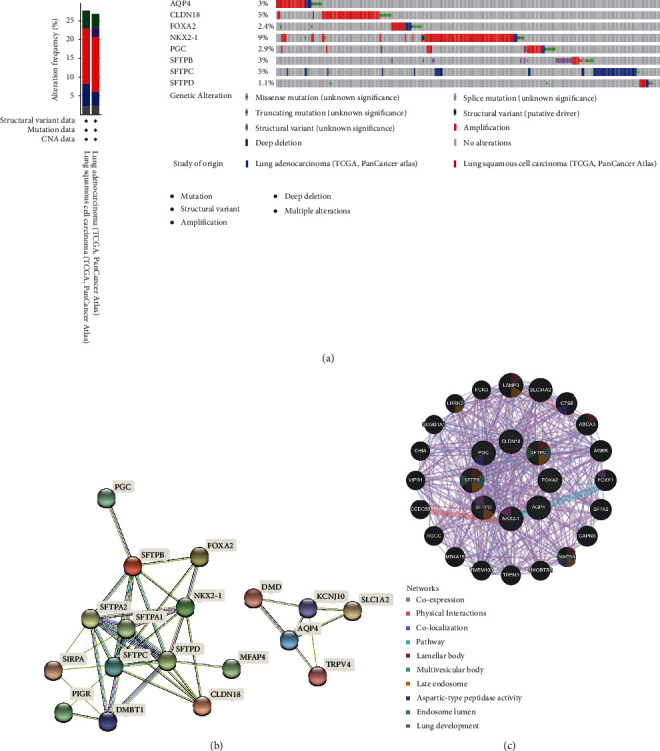
AT II-associated genes mutation in NSCLC and PPI analyses (cBioPortal and STRING). (a) Summary of alterations in AT II-associated genes in LUAD and LUSC. (b) and (c) PPI network and biological function of AT II-associated genes.

Epigenetic alteration plays a vital role in early malignancies, so a comprehensive analysis of the molecular characteristics of AT II-associated genes was further performed in the LUAD and LUSC samples, respectively. We used the cBioPortal online tool to analyze the AT II-associated genes alterations in LUAD (TCGA, Pan-Cancer Atlas) and LUSC (TCGA, Pan-Cancer Atlas). The results demonstrated that the alterations of 8 AT II-associated genes covered 273 samples out of 1053 patients with NSCLC (26%) (Figure 5(a)). Moreover, the mutation rates of AQP4, CLDN18, FOXA2, NKX2-1, PGC, SFTPB, SFTPC, and SFTPD were 3, 5, 2.4, 9, 2.8, 1.8, 5, and 1.1% of the investigated lung cancer samples, respectively (Figure 5(a)).

Moreover, a PPI network analysis of AT II-related genes was conducted with STRING. The results in Figure 5(b) illustrated that the DMBT1 gene which is a candidate tumor suppressor gene discovered in recent years was closely connected with AT II-associated genes (Figure 5(b)). Besides, some genes that play an important role in immune response regulation, blood cell proliferation, defense mechanisms, and acute phase response genes are also significantly connected with AT II-associated genes, including Microfibril-associated glycoprotein 4 (MFAP4*),* Pulmonary surfactant-associated protein A1(SFTPA1) (Figure 5(b)). The GeneMANIA results also revealed the functions of the differentially expressed AT II-associated genes, which including Leucine-rich repeat kinase 2 (LRRK2), lysosomal-associated membrane protein 3 (LAMP3), Cathepsin E (CTSE0), ATP-binding cassette transporter A3 (ABCA3), forkhead box F1 (FOXF1), and Napsin A (NAPSA), and these genes were mainly related to lung development, late endosome, aspartic-type peptidase activity. (Figure 5(c)).

### 3.5. Immune Cell Infiltration of AT II-associated genes in Patients With NSCLC

Immune cell level is associated with the proliferation and progression of the cancer cell. In this study, to investigate the relationship between AT II-associated genes and cancer-related inflammation and the infiltration of immune cells, we use the TIMER to reveal a comprehensive analysis of the correlation between eight AT II-associated genes and immune cell infiltration (Figure S3 and S4). ALL AT II-associated genes (including AQP4, FOXA2, NKX2-1, PGC, SFTPB, SFTPD, CLDN18, and SFTPC) were positively associated with the infiltration of six immune cell types (CD8+ T cells, B cells, CD4+ T cells, macrophages, neutrophils, and dendritic cells; all *p* < 0.05) in LUSC and positively associated with the infiltration of B cells in LUAD (*p* < 0.05). SFTPC and CLDN18 were positive connection with the infiltration of B cells and six immune cell types (CD8+ T cells, B cells, CD4+ T cells, macrophages, neutrophils, and dendritic cells) both in LUAD and LUSC cancers.

## 4. Discussion

The occurrence of lung cancer is a multistep process. For example, LUAD has always been thought to progress from atypical adenomatous hyperplasia (AAH) to adenocarcinoma in situ (AIS) [33]. Before the development of LUSC, we can observe preinvasive lesions in the airways [34]. Distinct molecular events and other malignant phenotypes make normal lung cells gain or lose certain functions leading to deregulation of key genetic signals involved in cell proliferation, differentiation, apoptosis, migration, and invasion [35–37]. Studies have shown that AT II cells can dedifferentiate into a cell stem-like state, which can continuously differentiate, proliferate, repair, and cause damage. Therefore, AT II is suspected to be the cell of origin in oncogene-driven lung cancers and can help maintain tumor progression [24] .

In recent years, 8 AT II-associated genes have been confirmed to play key roles in tumor growth and development. For example, FOXA2 has been proved that it plays crucial roles in lung morphogenesis, surfactant protein production, goblet cell differentiation, and mucin expression [38]. Besides, Liu et al. experimentally found that the histone demethylase PHF8 can drive neuroendocrine prostate cancer (NEPC) development by epigenetically upregulation of FOXA2 [39]. Thyroid transcription factor 1 (TTF-1 or NKX2-1) has been known as an important development regulator of driving the brain, lungs, and thyroid maturation and morphogenesis [40]. Studies have demonstrated that NKX2-1 gene mutations related to compensated congenital hypothyroidism and unexplained respiratory distress due to lung hypoplasia in neonates [41]. NKX2-1 amplification and overexpression also have been proved to have contributed to lung cancer cell proliferation rates and survival results [42]. Interestingly, some researchers found an opposite phenomenon that NKX2-1 can constrain lung adenocarcinoma in part by repressing the embryonically restricted chromatin regulator Hmga2 [43]. Thus, the oncogenic and inhibitory function of NKX2-1 in the same tumor type confirms its role as a bifunctional lineage factor. Aquaporins (AQPs) are water channel proteins that can be capable of selectively transporting water and other small solutes across cells [44, 45]. In the lung, AQPs were supposed to facilitate fluid transportation in alveolar space, airway humidification, pleural fluid absorption, and submucosal gland secretion. AQP4 is one of members of the aquaporin family which was first discovered in 1994 [45, 46]. The change of AQP4 expression is associated with many central nervous system (CNS) diseases including epilepsy, edema, stroke, and glioblastoma [47]. Besides, in breast cancer, thyroid carcinoma (undifferentiated), and stomach cancer, the expression of AQP4 is low [48–51]. On the contrary, studies found that AQP4 is highly expressed in lung cancer and is involved in the invasion of lung cancer cells [52, 53]. Surfactant proteins (SP) are involved in surfactant function and innate immunity in the human lung. In cystic fibrosis (CF), the genetic contribution of the surfactant protein genes, SFTPB, SFTPC, and SFTPD are contained [54]. Finally, CLDN18 is required for intercellular connectivity and has been reported to be involved in cell migration and metastasis, making it an oncogene in various cancer types, including pancreatic, esophageal, ovarian, and lung cancer [55].

In this study, we first systematically analyzed the expression of eight AT II-associated genes (AQP4, SFTPB, SFTPC, SFTPD, CLDN18, FOXA2, NKX2-1, and PGC) in lung cancer. The expression levels of the eight genes in lung cancer were lower. Additionally, we also verified that the expression of AT II-associated genes was observably related to clinical cancer stages in NSCLC patients. These results indicate that all these eight AT II-associated genes might take a significant part in the tumorigenesis and progression of NSCLC. Besides, all these eight AT II-associated genes were found to be notably related to OS in lung cancer patients, and low-expression was associated with short OS in lung cancer patients. Seven genes except AQP4 were significantly positive associated with FP. And low-expression of CLDN18, FOXA2, and SFTPD apparently led to a short PPS. All these results indicate that AT II-associated genes might be a protectable factor for survivals of NSCLC patients and thus might be potential prognostic biomarkers. In addition, our study showed that the expression level of AT II-associated genes was significantly correlated with the infiltration of six immune cell types. This result also suggests that AT II-associated genes may also reflect the immune status besides the disease prognosis.

## 5. Conclusion

In conclusion, this study provided clues of the values of AT II-associated genes (AQP4, SFTPB, SFTPC, SFTPD, CLDN18, FOXA2, NKX2-1, and PGC) as clinical biomarkers and therapeutic targets in NSCLC. We believe that these eight AT II-associated genes were expected to become new prognostic biomarkers in NSCLC and provide some new inspirations to assist in the design of new immunotherapies.

## Figures and Tables

**Table 1:** The relationship between the expression level of AT II-associated genes and NSCLC prognosis.

AT II-associated genes	Kaplan-Meier plotter (log rank *p*)			GEPIA (log rank *p*)
	OS	FP	PPS	DFS
AQP4	2.40E-04	5.60E-02	4.00E-01	7.40E-01
CLDN18	1.90E-05	9.10E-04	3.20E-02	6.90E-01
FOXA2	1.60E-12	6.70E-05	2.10E-02	8.10E-01
NKX2-1	4.90E-10	3.10E-02	1.20E-01	1.30E-01
PGC	1.00E-08	2.40E-04	5.90E-01	2.20E-01
SFTP-B	6.30E-10	4.80E-02	3.80E-01	4.20E-01
SFTPC	1.40E-03	4.00E-02	1.10E-01	2.90E-01
SFTPD	1.60E-10	6.20E-05	2.10E-02	4.00E-01

Note: OS: overall survival; FP: progression-free survival; PPS: postprogression survival; DFS: disease-free survival.

## Data Availability

All data generated or analyzed during this study are included in this published article.

## Supplementary Materials

Supplementary 1
*Figure S1*. The relative expression level of AT II-associated genes in LUAD and LUSC (GEPIA). The darker the color of the bar, the higher the relative expression. The result evaluated that SFTPB was the highest expression in both LUAD and LUSC.

Supplementary 2
*Figure S2*. Prognostic value of AT II-associated genes (SFTPB, SFTPC, SFTPD, and PGC) in LUAD and LUSC (Kaplan-Meier plotter).

Supplementary 3
*Figure S3*. Correlations between AT II-associated genes and immune cell infiltration (TIMER). Correlations between the abundance of immune cells and the expression of AQP4, CLDN18, FOXA2, NKX2-1, PGC, SFTPB, SFTPC, and SFTPD in LUAD.

Supplementary 4
*Figure S4*. Correlations between AT II-associated genes and immune cell infiltration (TIMER). Correlations between the abundance of immune cells and the expression of AQP4, CLDN18, FOXA2, NKX2-1, PGC, SFTPB, SFTPC, and SFTPD in LUSC.
