# Supplementary figures and images for: Comprehensive Analysis of the Molecular Characteristics and Prognosis value of AT II-associated Genes in Non-small Cell Lung Cancer
Source: Comput Math Methods Med. 2022 Sep 26;2022:3106688. doi: 10.1155/2022/3106688 (PMC9530922; doi:10.1155/2022/3106688)

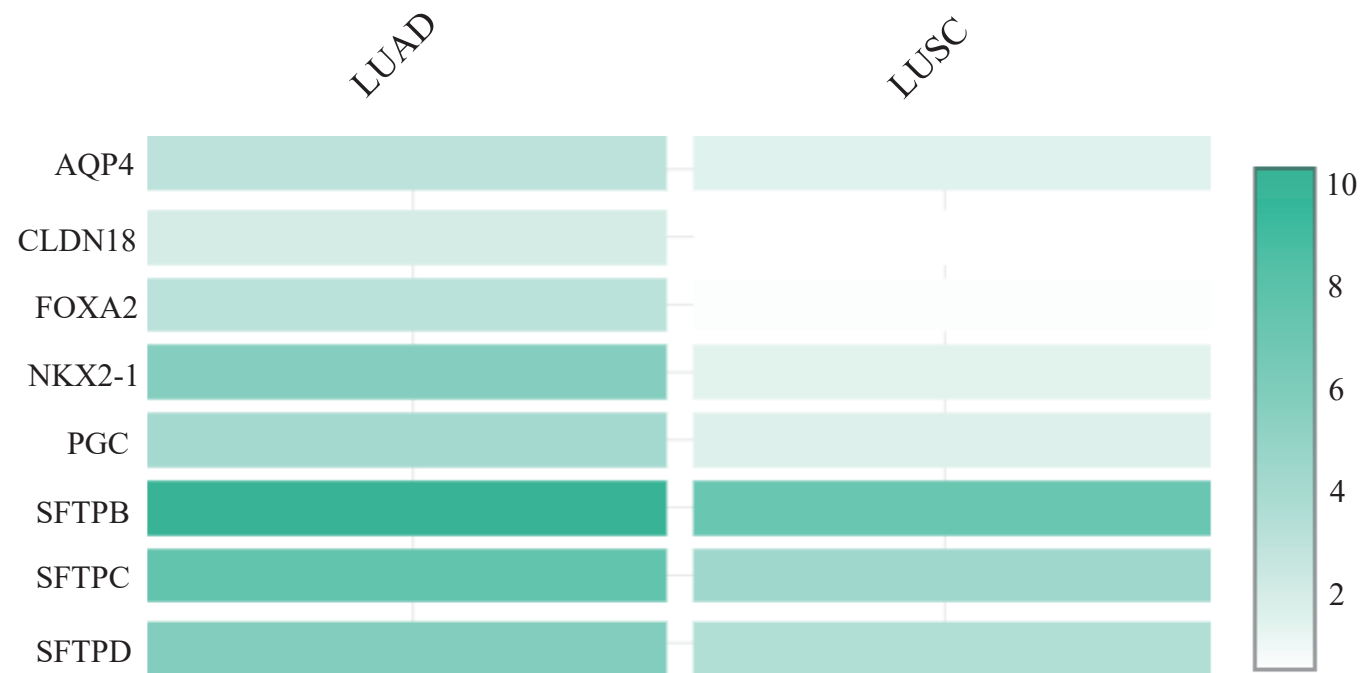

Supplement: Supplementary 1 — Figure S1. The relative expression level of AT II-associated genes in LUAD and LUSC (GEPIA). The darker the color of the bar, the higher the relative expression. The result evaluated that SFTPB was the highest expression in both LUAD and LUSC. [file 3106688.f1.pdf]

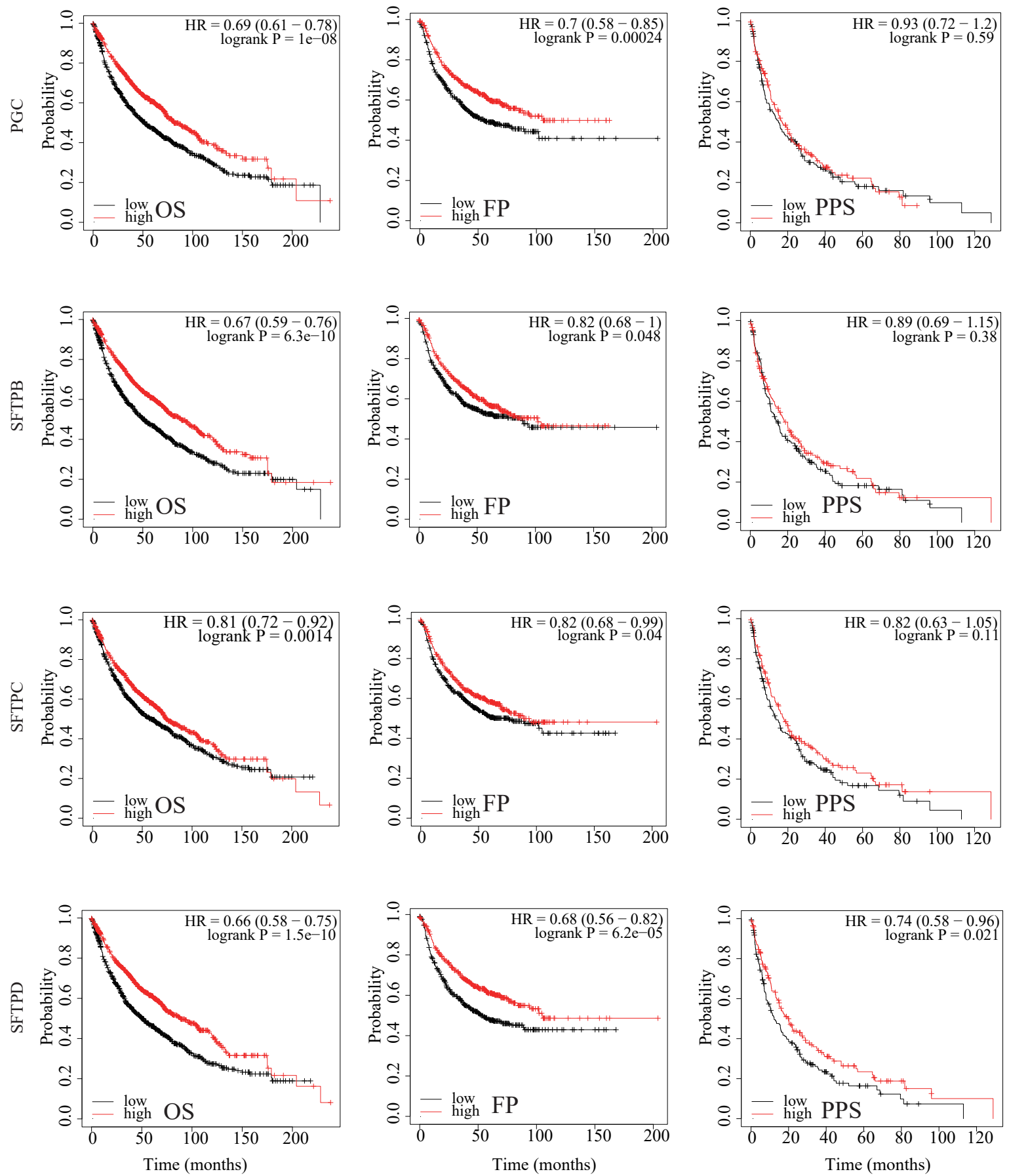

Supplement: Supplementary 2 — Figure S2. Prognostic value of AT II-associated genes (SFTPB, SFTPC, SFTPD, and PGC) in LUAD and LUSC (Kaplan-Meier plotter). [file 3106688.f2.pdf]

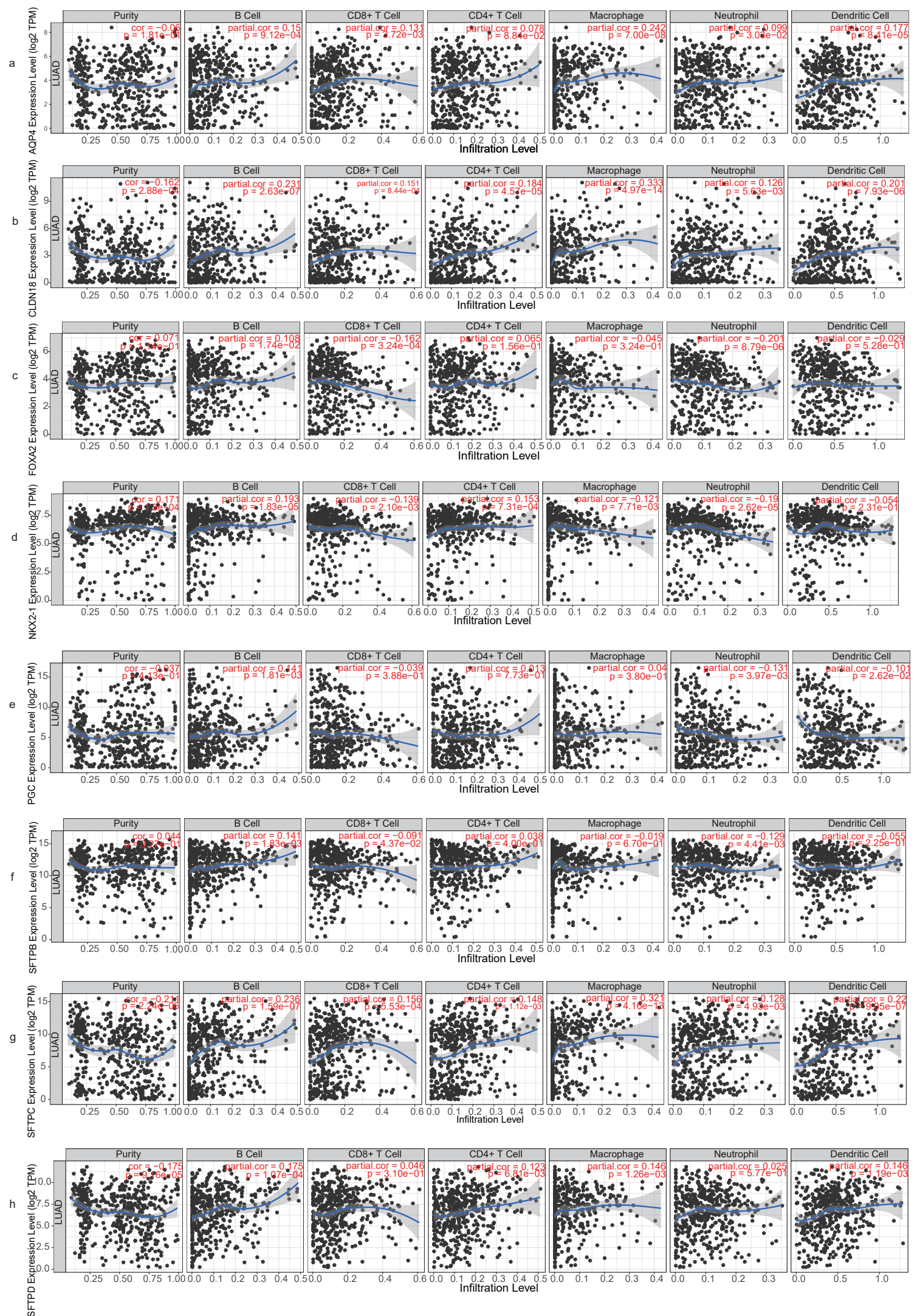

Supplement: Supplementary 3 — Figure S3. Correlations between AT II-associated genes and immune cell infiltration (TIMER). Correlations between the abundance of immune cells and the expression of AQP4, CLDN18, FOXA2, NKX2-1, PGC, SFTPB, SFTPC, and SFTPD in LUAD. [file 3106688.f3.pdf]

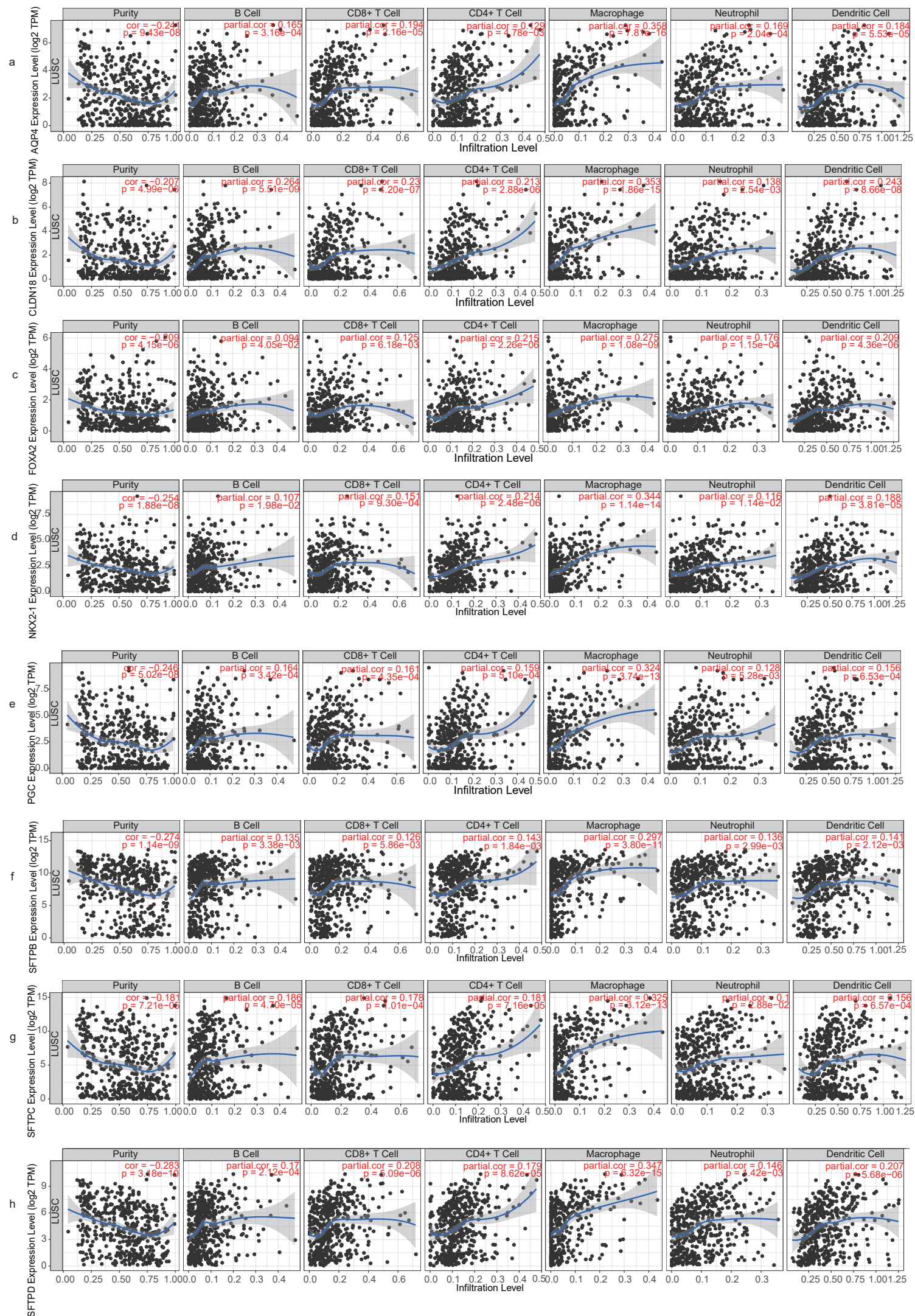

Supplement: Supplementary 4 — Figure S4. Correlations between AT II-associated genes and immune cell infiltration (TIMER). Correlations between the abundance of immune cells and the expression of AQP4, CLDN18, FOXA2, NKX2-1, PGC, SFTPB, SFTPC, and SFTPD in LUSC. [file 3106688.f4.pdf]
